# Evaluation of the efficacy and treatment-emergent adverse events of deuruxolitinib for moderate to severe alopecia areata: a dose-ranging meta-analysis of 1,372 randomized patients

**DOI:** 10.3389/fmed.2025.1641245

**Published:** 2025-10-07

**Authors:** Mulham Kalantan, Bader Bashrahil, Abdulaziz Aljuaid, Hassan Bogari, Sahal Samarkandy, Abdulhadi Jfri

**Affiliations:** ^1^College of Medicine, King Saud bin Abdulaziz University for Health Sciences, Jeddah, Saudi Arabia; ^2^King Abdullah International Medical Research Center, Jeddah, Saudi Arabia; ^3^Department of Dermatology, Ministry of the National Guard-Health Affairs, Jeddah, Saudi Arabia; ^4^Department of Dermatology, King Fahad Armed Forces Hospital, Jeddah, Saudi Arabia

**Keywords:** alopecia, alopecia aerata (AA), biologic, deuruxolitinib, CTP-543, JAK inhibitor

## Abstract

**Introduction:**

Alopecia areata (AA) is an immune disease characterized by non-scarring hair loss. With the increasing use of Janus kinase (JAK) inhibitors in immune-related conditions, their potential role in AA treatment is gaining attention. Deuruxolitinib has emerged as a potential treatment for moderate to severe AA. This is the first systematic review and meta-analysis that aims to assess the efficacy of deuruxolitinib in moderate to severe AA.

**Methods:**

We systematically searched Cochrane Central Register of Controlled Trials (CENTRAL), Medline, and ClinicalTrials.gov for relevant data. Deuruxolitinib vs. placebo was evaluated, and efficacy was measured using severity of alopecia tool (SALT) and Hair Satisfaction Participants Reported Outcome (SPRO), with the primary time point of assessment at week 24. Treatment-emergent adverse events (TEAEs) such as increased creatinine kinase (CPK), acne, and headache were specifically assessed at week 28. Effect sizes were presented using mean difference (MD) or risk ratio (RR). Statistical heterogeneity was measured by *I*^2^, with a 95% confidence interval (CI) and *p*-value less than 0.05 considered significant. Risk of bias was assessed using the Revised Cochrane risk of bias tool. Subgroup analysis was conducted for different regimens (8 mg and 12 mg) and TEAEs of interest. This research was registered in PROSPERO (CRD42023417104).

**Results:**

Three randomized controlled trials involving 1,372 patients were included. Deuruxolitinib demonstrated a significant improvement in SALT score change from baseline [MD = −47.26, 95% CI = (−53.47, −41.05), *p* < 0.00001, *I*^2^ = 76%], with a significant number of patients achieving 75% [RR = 93.66, 95% CI = (23.42, 374.65), *p* < 0.00001, *I*^2^ = 0%] and 90% [RR = 65.26, 95% CI = (16.28, 261.58), *p* < 0.00001, *I*^2^ = 0%] improvement from baseline. Patients randomized to deuruxolitinib reported a significant improvement in SPRO [MD = −1.52, 95% CI = (−1.76, −1.27), *p* < 0.00001, *I*^2^ = 69%], with many experiencing more than two points of improvement [RR = 4.98, 95% CI = (3.79, 6.54), *p* < 0.00001, *I*^2^ = 0%]. TEAEs included elevated CPK levels [RR = 2.79, 95% CI = (1.5, 4.99), *p* = 0.0006, *I*^2^ = 0%], headaches [RR = 1.49, 95% CI = (0.98, 6.54), *p* = 0.06, *I*^2^ = 44%], and acne (significant in the 12 mg dose only) [RR = 1.80, 95% CI = (0.84, 3.88), *p* = 0.13, *I*^2^ = 64%].

**Discussion:**

In conclusion, deuruxolitinib shows promising efficacy in treating moderate to severe AA, leading to significant improvements in hair regrowth and patient-reported satisfaction. While certain TEAEs such as increased CPK levels, headaches, and acne (especially at the 12 mg dose), they were generally manageable. Further research and vigilant monitoring for long term safety are necessary before widespread adoption of deuruxolitinib for AA treatment.

## Introduction

Alopecia areata (AA), an autoimmune condition that affects both men and women and which is characterized by patches of non-scarring alopecia on the scalp, face, and body hair, is clinically heterogeneous. Approximately 0.6–2.0% of new cases in dermatology clinics in the US and the UK are patients with AA: It is a condition frequently seen in clinical settings (1, 2). The incidence risk of AA has been estimated to range from 0.57 to 3.8% in hospital-based studies conducted globally (3). AA frequently manifests as a cyclical disorder with fluctuating levels or patterns of hair loss as well as unpredictable periods of hair loss and spontaneous regrowth (4). The most common forms of AA include small patches of hair loss limited to one or more discrete, well-circumscribed areas that are round or oval and located on the scalp or body; or complete loss of scalp hair [Alopecia Totalis (AT)]; or complete loss of scalp, facial, and body hair [Alopecia Universalis (AU)] (5). According to reports, the first onset appears by age 40 in more than 80% of patients and by age 20 in 40% (3). Despite AA patients frequently experiencing remission within the first year, an estimated 4.5–36.1% of patients eventually advance to develop AT and/or AU (AT/AU). AA has a significant negative impact on health-related quality of life and social functioning for both patients and their family members and is associated with high rates of depression and anxiety (4).

The pathophysiology of AA involves increased levels of interferon-gamma (IFN-γ) and the common gamma chain (γc) cytokines interleukin (IL)-2, IL-7, and IL-15 which promote cytotoxic CD8+NKG2D+ T cells adjoining hair follicles and attacking them (6). IFN-γ and γc cytokines bind to their receptors and activate Janus kinase-mediated signaling, which results in the phosphorylation of signal transducers and activation of transcription (STAT) molecules (6–8). The Janus kinase receptors JAK 1/2 and JAK 1/3, as well as activation of transcription (STAT), trigger an additional cellular immune response that ultimately increases the production of IFN-γ and IL-15 in hair follicles (6).

The age of the patient, as well as the severity and impact of hair loss, are typically taken into consideration when choosing therapy for AA patients (6). Clinically, there is a wide range of therapeutic options. For patients with mild and patchy AA, intralesional and topical corticosteroids are considered as first-line treatment (9). For more severe involvement, systemic therapy including corticosteroids, methotrexate, azathioprine, or cyclosporine may be considered to alleviate AA (4, 6, 10). The choice and effectiveness of corticosteroid therapy are influenced by factors such as dosage, duration, and route of administration (oral, intramuscular, or intravenous), and may be associated with adverse effects including weight gain, acne, and endocrine disturbances (10). While some patients achieve prolonged remission, data quality remains limited and often derives from case reports and small series. Therefore, the treatment of AA urgently requires more effective and safe therapeutic methods.

According to recent genome-wide association studies and preclinical studies, Janus kinase/signal transducers and activators of the transcription pathway play a crucial role in AA (7, 11, 12). These investigations led the way for the development of Janus kinase inhibitors as an AA treatment (13). Janus kinase inhibitors, a group of small-molecule drugs, can suppress one or more intracellular tyrosine kinases in the JAK–STAT signaling pathway, including JAK1, JAK2, JAK3, and tyrosine kinase 2 (TYK2) (10). They induce immune suppression by targeting various cytokines and inflammatory pathways, including IL-2, IL-7, IL-15, IL-21, and IFN-γ. All of these appear to be involved in the pathogenesis of AA (6, 10). According to numerous clinical trials, the use of JAK inhibitors in the treatment of AA has resulted in satisfactory outcomes with a tolerable side effect profile (10). A recent expert consensus, the alopecia areata consensus of experts (ACE) study, also emphasized the promising role of JAK inhibitors in the treatment of moderate-to-severe AA, recommending their use especially for patients with more than 50% scalp hair loss who are unresponsive to conventional therapies (14). Baricitinib, a JAK1/2 inhibitor, is the first drug among JAK inhibitors to be approved by the Food and Drug Administration (FDA) as a treatment for AA (15). Ritlecitinib (JAK3/TEC kinase inhibitor) has also been recently approved by both the FDA and the European Medicines Agency (EMA), expanding the range of regulated options (16). Tofacitinib (JAK 1/3 inhibitor), ruxolitinib (JAK 1/2 inhibitor), and brepocitinib (JAK1/TYK2) have been used off-label to treat AA as they show efficacy and are well tolerated (17, 18). Deuruxolitinib, CTP-543, is a deuterium—modified analog of ruxolitinib that selectively inhibits JAK1 and JAK2. It is under development for the treatment of moderate-to-severe AA and has shown a promising results in phase III trials. This systematic review and meta-analysis aimed to assess the efficacy and safety of deuruxolitinib in moderate-to-severe AA.

## Methods

This research was registered before conducting an initial search in accordance with PROSPERO (CRD42023417104.) This article utilized the preferred reporting items for systematic reviews and meta-analysis (PRISMA) checklist. As this study is a secondary analysis of previously published data, ethical approval was not required.

**Figure 1:**
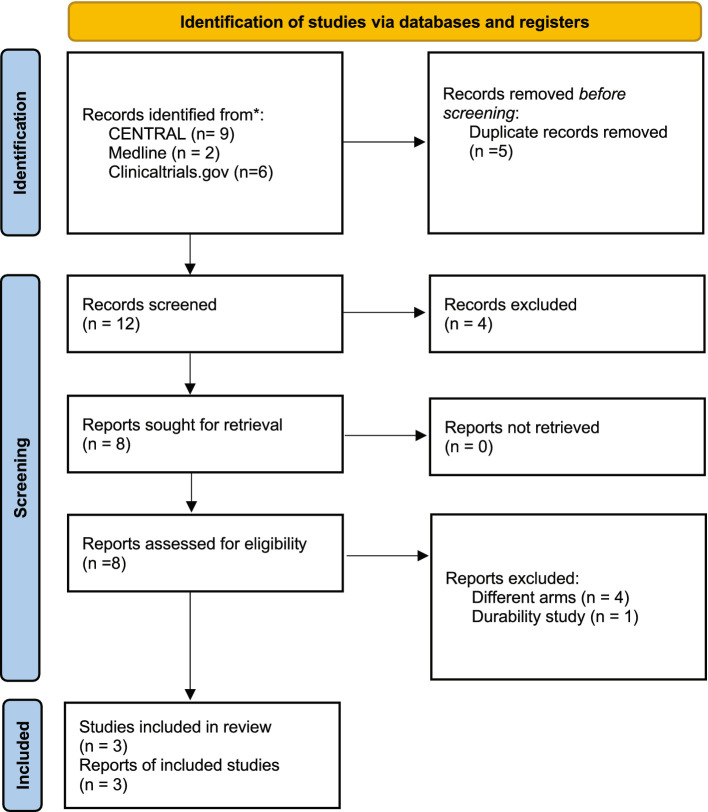
Flowchart of the selection process.

**Figure 2:**
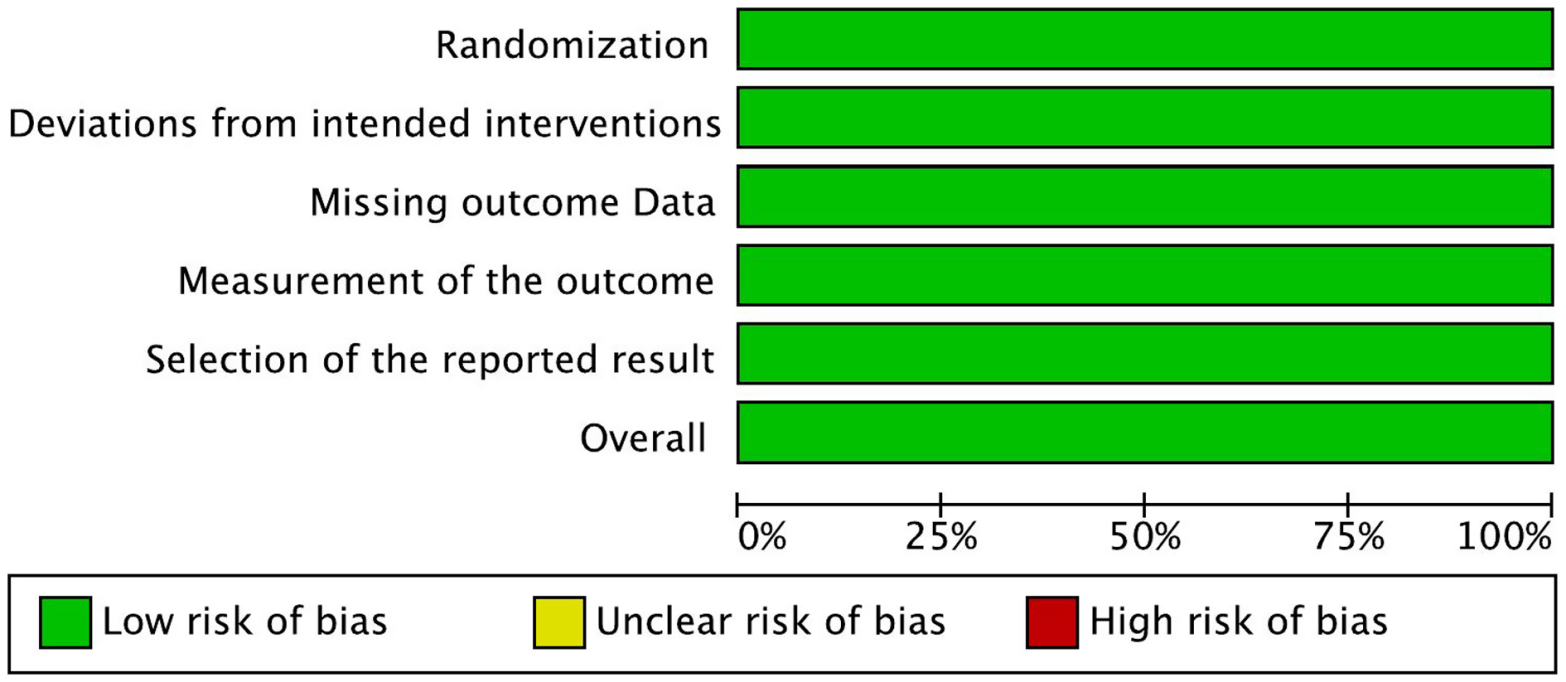
Risk of bias graph.

**Figure 3:**
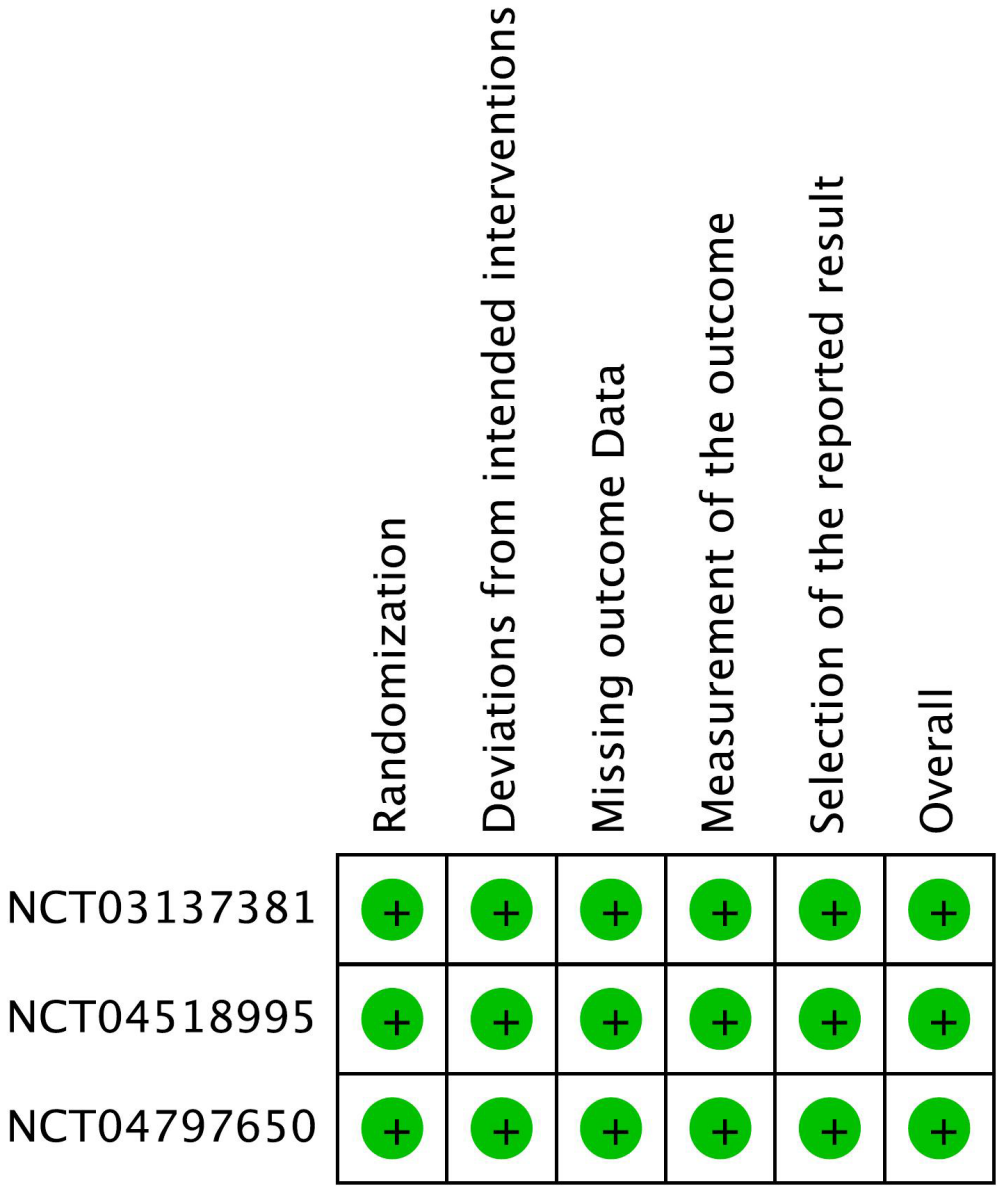
Risk of bias summary.

**Figure 4:**
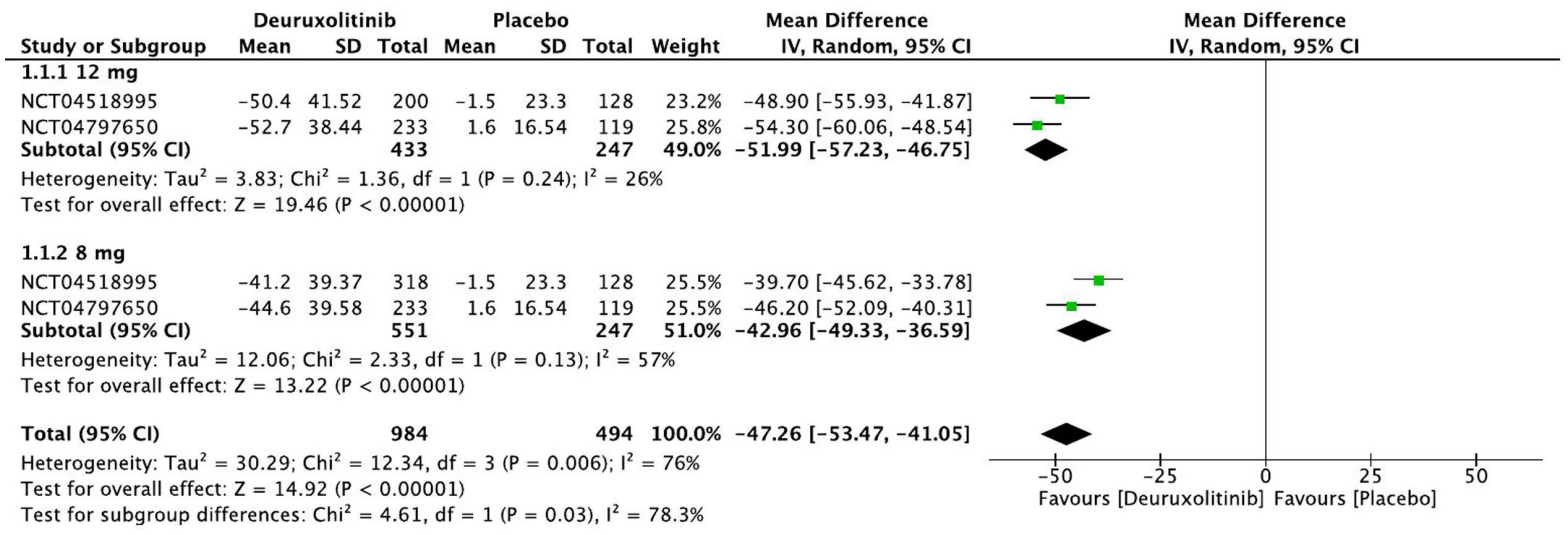
Forest plot of Relative change in SALT score from baseline.

**Figure 5:**
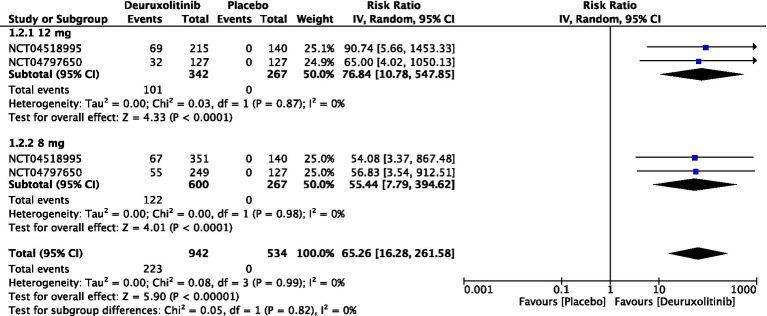
Forest plot of 90% reduction in SALT score.

**Figure 6:**
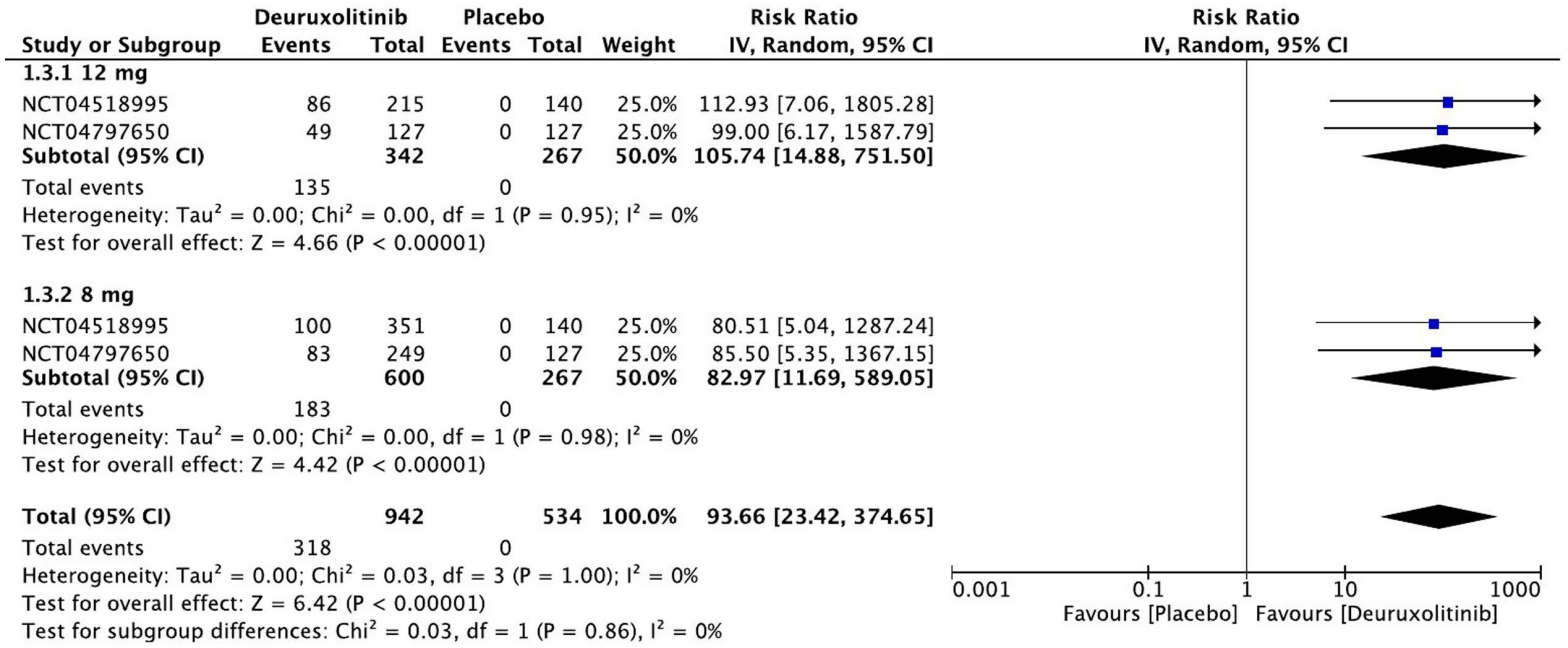
Forest plot of 75% reduction in SALT score.

**Figure 7:**
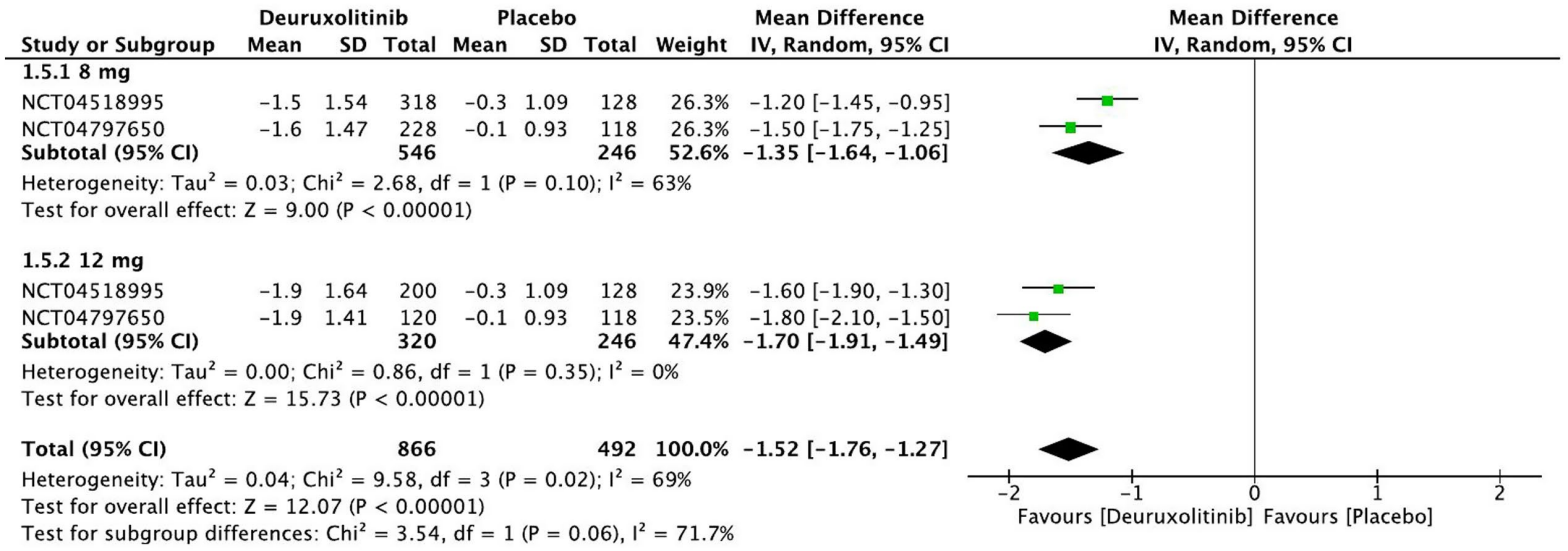
Forest plot of SPRO changes from baseline.

**Figure 8:**
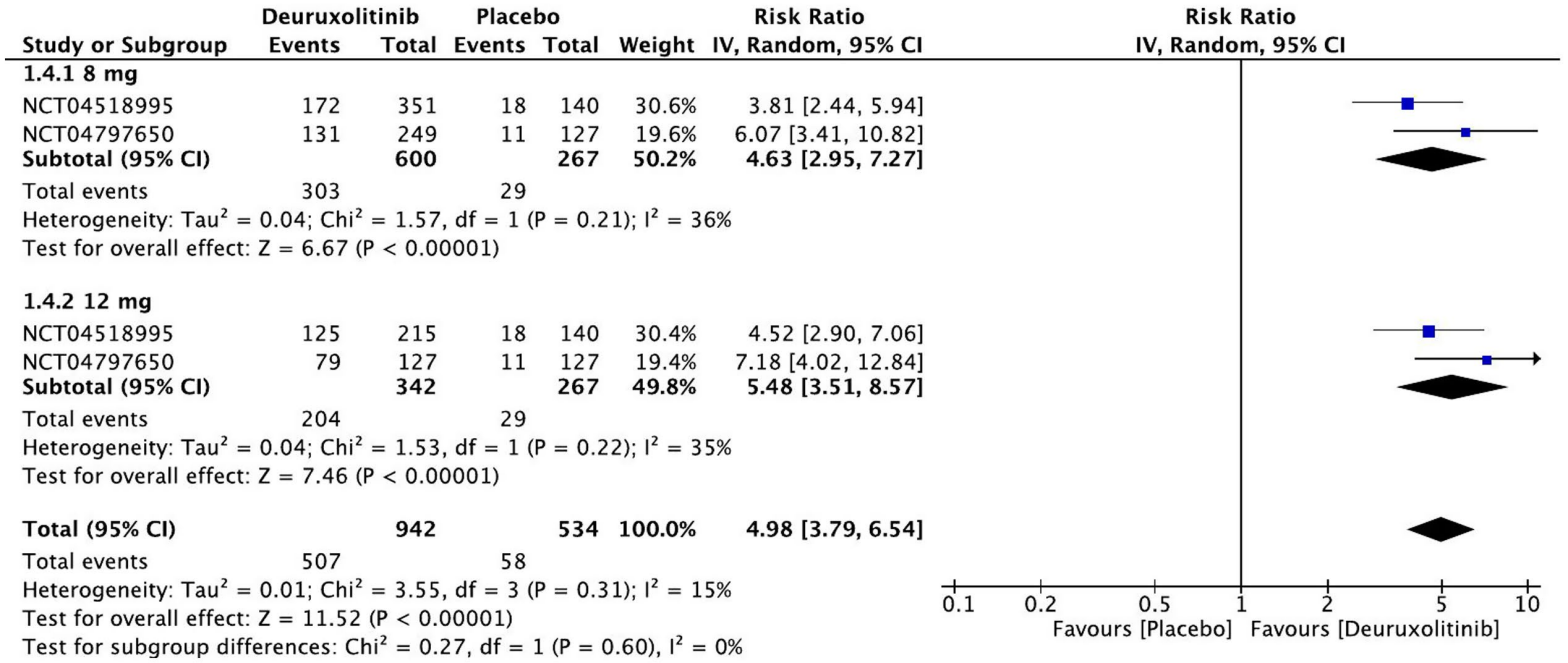
Forest plot of >2 points in SPRO.

**Figure 9:**
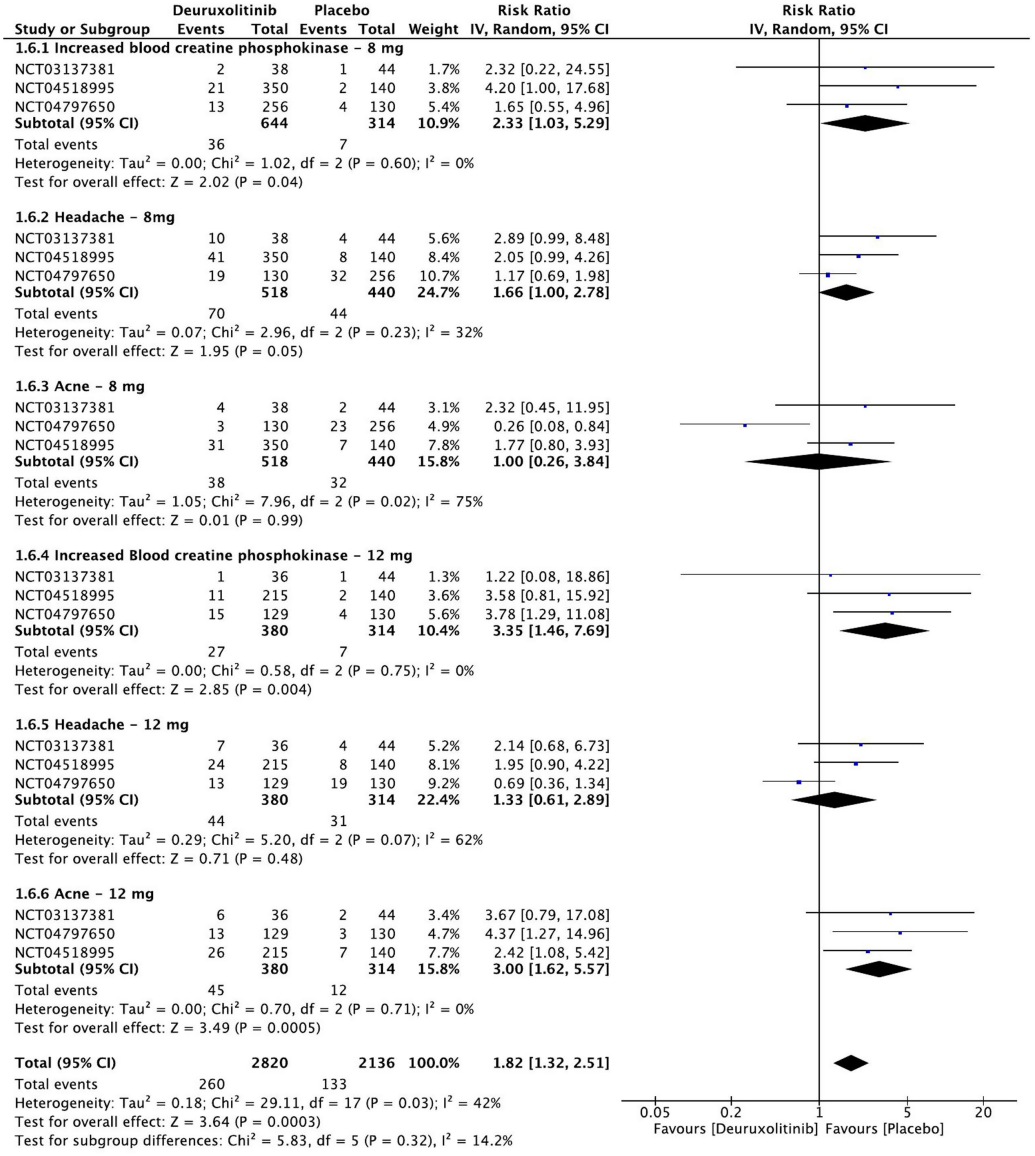
Forest plot of Treatment-emergent adverse events of interest.

**Figure 10:**
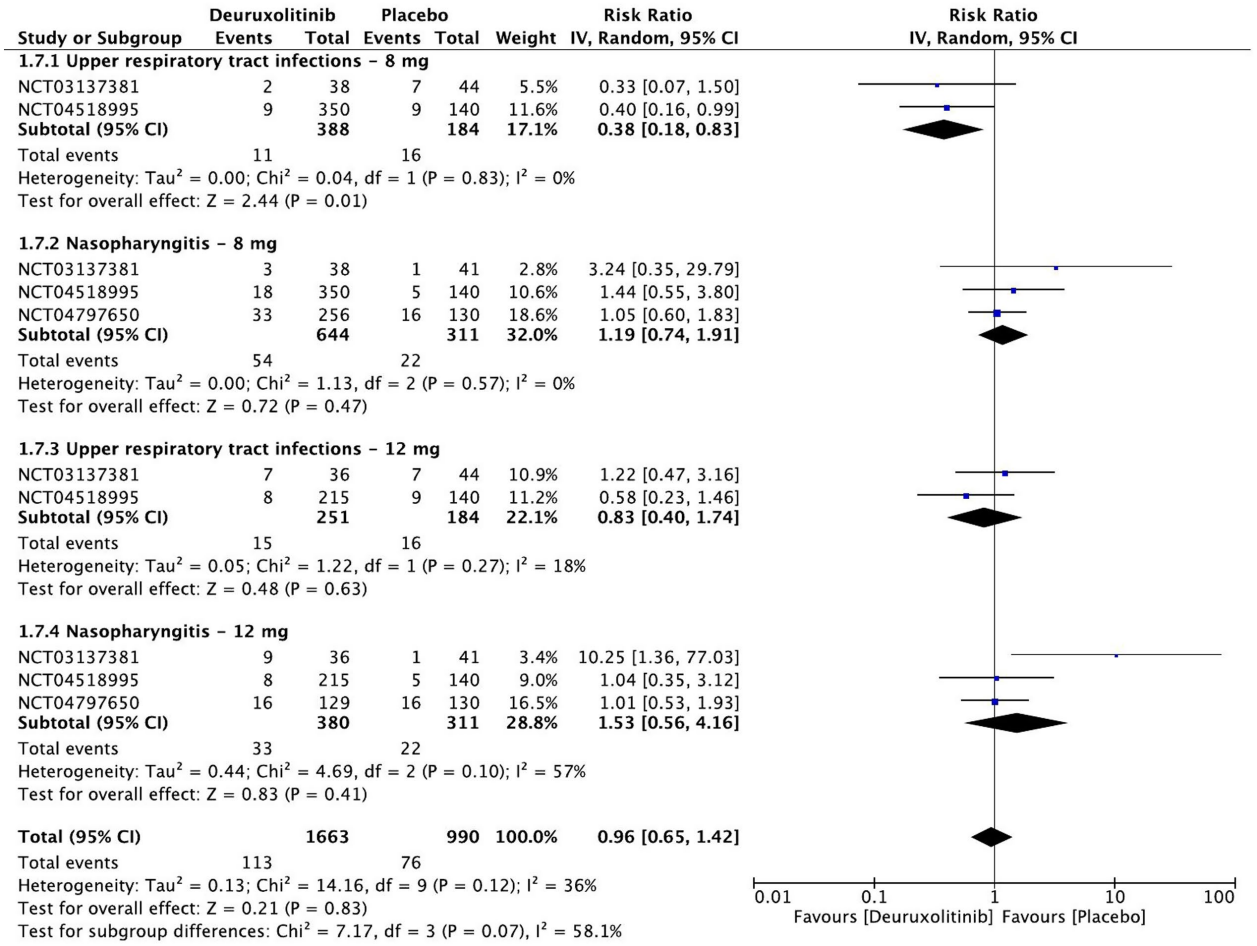
Forest plot of infections and infestations.

### Eligibility criteria

This meta-analysis included only randomized clinical trials (RCTs) that compared the efficacy of deuruxolitinib versus placebo in patients with moderate-to-severe AA who are between 18 and 65 years of age and were in active episodes of hair loss lasting ≥6 months and not exceeding 10 years. Eligible studies had patients with ≥50% hair loss, as measured by the severity of alopecia tool (SALT), at screening and who were not being treated for AA or receiving other treatments that might have affected hair regrowth or immune response. Excluded patients were those who received any systemic immunosuppressive medications within 3 months of screening or any biologic medications within 6 months of screening.

### Search strategy

A systematic search was conducted using Cochrane Central Register of Controlled Trials (CENTRAL), Medline, and ClinicalTrials.gov databases from their initiation to 30 July 2023. The search aimed to identify relevant data regarding the use of deuruxolitinib in the treatment of moderate-to-severe AA. The strategy employed appropriate keywords and mesh terms related to AA and deuruxolitinib. No publication date restriction was applied. The complete search strategies for each database are provided in the Supplementary file.

### Study selection and data extraction

The selection process involved screening studies based on eligibility criteria. Titles and abstracts were assessed for relevance and duplicates were removed. Full-text articles were obtained for potentially eligible studies and further evaluated for inclusion. Data extraction was performed for the included studies and key information such as study characteristics, participant demographics, intervention details, outcome measures, and treatment-emergent adverse events (TEAEs) were extracted using standardized forms. Data extraction was conducted independently by two reviewers, and any disparities in the extracted data were diligently addressed and resolved by a third author’s opinion.

### Outcomes

The efficacy outcomes of this meta-analysis involved the percentage of change from baseline in SALT score, the proportion of patients achieving 75 and 90% improvement in SALT score, the change in Hair Satisfaction Participants Reported Outcome (SPRO) scale from baseline, and the proportion of participants achieving ≥2 point change from baseline in SPRO scale. Week 24 was the time point of assessment for efficacy outcomes, while safety was evaluated at week 28. The safety profile of deuruxolitinib was assessed by measuring the incidence of TEAEs including increased creatinine kinase (CPK), acne, and headache that were evaluated specifically in addition to infections and infestations.

### Meta-analysis

For the Data analysis, RevMan version 5.3 was performed, and statistical analysis was conducted using the random-effects model. Effect sizes were calculated using mean difference (MD) for continuous outcomes (SALT score change from baseline, SPRO change from baseline) and risk ratio (RR) for dichotomous outcomes (75 and 90% improvement from baseline, >2 points in SPRO, TEAEs). Statistical heterogeneity was assessed using the *I*^2^, with a 95% confidence interval (CI) and a *p*-value less than 0.05 considered statistically significant. A subgroup analysis was conducted to explore different deuruxolitinib regimens and specific TEAEs of interest. The first regimen included patients who were given 12 mg of deuruxolitinib twice daily (BID). The second regimen included patients who were given an 8 mg dose BID. The quality and certainty of evidence were evaluated using the Grading of Recommendations Assessment, Development, and Evaluation (GRADE) criteria.

### Risk of Bias assessment

The Revised Cochrane Risk of Bias tool (RoB 2) was used to assess the risk of bias in the studies included (19). This tool evaluates random sequence generation, allocation concealment, blinding, incomplete outcome data, selective outcome reporting, and other sources of bias. The risk of bias in the reviewed RCTs was evaluated by two reviewers independently. A review process was used to categorize individual studies as low risk of bias, some concerns, or high risk of bias. The reviewers discussed and worked out their differences before coming to a final verdict and any disagreement would be resolved by discussion with a third reviewer. Assessment of reporting bias (e.g., funnel plots or Egger’s test) was not conducted due to the small number of included studies (*n* < 10), which limits the reliability and interpretability of such analyses. This is consistent with current methodological recommendations.

## Results

A total of 17 studies were identified initially after the database search, of which five were duplicated across the selected databases and excluded from this study. Twelve potential related articles were identified for screening. In the screening process after reading titles and abstracts, four articles were excluded. The full text of the remaining eight articles was assessed, and articles with incomplete results or different arms were excluded. Eventually, three randomized clinical trials (NCT03137381, NCT04518995, NCT04797650) (20) that matched our inclusion criteria were included in this study (Figure 1)

### Baseline characteristics

The three randomized controlled trials assessed 1,372 participants of three different arms (deuruxolitinib 8 mg BID, deuruxolitinib 12 mg BID, and placebo). The baseline characteristics of the included studies are described in Table 1. Among the included three RCTs, the numbers of participants in deuruxolitinib 8 mg and 12 mg BID were 647 and 381 respectively, while the placebo arm comprised 314 participants. The mean age of participants ranged from 35.7 to 39.7. Of 1,372 participants, the majority were female (891, 65%), while men comprised 481 (35%).

**Table 1:** Characteristics of included trials.

Study name	Study design	Trial follow-up period	Study regimens (sample size)	Mean age (SD)	Gender	Race	Baseline SALT
NCT03137381 King et al. (18, 20)	RCT	24 weeks	Deuruxolitinib 4 mg BID (*n* = 30)	35.7 (11.01)	Males; *n* = 8, females; *n* = 22	White; *n* = 25, Black *n* = 2, Asian; *n* = 2, other; *n* = 1	88.8
			Deuruxolitinib 8 mg BID (*n* = 38)	37.3 (14.18)	Males; *n* = 12, females; *n* = 26	White; *n* = 25, Black *n* = 7, Asian; *n* = 2, other; *n* = 3	89.1
			Deuruxolitinib 12 mg BID (*n* = 37)	35.8 (12.37)	Males; *n* = 9, females; *n* = 28	White; *n* = 30, Black *n* = 3, Asian; *n* = 4	87.3
			Placebo tablets BID (*n* = 40)	37.8 (13.50)	Males; *n* = 15, females; *n* = 29	White; *n* = 33, Black *n* = 7, Asian; *n* = 2, Pacific islander; *n* = 1, other; *n* = 1	86.8
NCT04518995 (THRIVE-AA1)	RCT	24 weeks	Deuruxolitinib 8 mg BID (*n* = 351)	38.9 (13.32)	Males; *n* = 134, females; *n* = 217	White; *n* = 241, Black *n* = 40, Asian; *n* = 22, Pacific islander; *n* = 3, American Indian or Alaska native; *n* = 2, other; *n* = 17, not reported; *n* = 26	85.5
			Deuruxolitinib 12 mg BID (*n* = 215)	38.2 (12.80)	Males; *n* = 84, females; *n* = 131	White; *n* = 145, Black *n* = 27, Asian; *n* = 21, Pacific islander; *n* = 1, American Indian or Alaska native; *n* = 1, other; *n* = 6, not reported; *n* = 14	85.2
			Placebo tablets BID (*n* = 140)	38.7 (13.81)	Males; *n* = 51, females; *n* = 89	White; *n* = 98, Black *n* = 16, Asian; *n* = 10, Pacific islander; *n* = 1, other; *n* = 5, not reported; *n* = 10	88.1
NCT04797650 (THRIVE-AA2)	RCT	24 weeks	Deuruxolitinib 8 mg BID (*n* = 258)	38.4 (12.30)	Males; *n* = 81, females; *n* = 177	White; *n* = 100, Black *n* = 10, Asian; *n* = 7, American Indian or Alaska native; *n* = 1, other; *n* = 1, not reported; *n* = 11	88.1
			Deuruxolitinib 12 mg BID (*n* = 129)	39.0 (12.49)	Males; *n* = 84, females; *n* = 45	White; *n* = 109, Black *n* = 7, Asian; *n* = 4, not reported; *n* = 28	86.7
			Placebo tablets BID (*n* = 130)	39.7 (12.49)	Males; *n* = 42, females; *n* = 88	White; *n* = 100, Black *n* = 10, Asian; *n* = 7, American Indian or Alaska native; *n* = 1, other; *n* = 1, not reported; *n* = 11	88.9

RCT, randomized controlled trial; SD, standard deviation; BID, twice daily; SALT, severity of alopecia tool score.

### Risk of Bias

The risk of bias in the reviewed RCTs was evaluated and assessed. All three included RCTs showed low risk of bias in all five domains (Figures 2, 3).

### Efficacy outcomes

#### Relative change in SALT score from baseline

Two studies representing 1,231 patients evaluated the percentage of SALT score change from baseline up to week 24. Cumulatively, deuruxolitinib demonstrated a significant improvement in SALT score change from baseline compared to placebo [MD = −47.26, 95% CI = (−53.47, −41.05), *p* < 0.00001, *I*^2^ = 76%]. Both high dose (12 mg BID) and low dose (8 mg BID) demonstrated remarkable decreases in SALT score versus placebo, respectively, [MD = −51.99, 95% CI = (−57.23, −46.75), *p* < 0.00001, *I*^2^ = 26%] [MD = −42.96, 95% CI = (−49.33, −36.59), *p* < 0.00001, *I*^2^ = 57%]. (High certainty of evidence) (Figure 4).

#### 90% reduction in SALT score

One thousand two hundred and nine patients from two studies measured the number of participants who achieved a reduction in SALT score of at least 90% from baseline to week 24 [RR = 65.26, 95% CI = (16.28, 261.58), *p* < 0.00001, *I*^2^ = 0%]. The high dose showed the most significant improvement [RR = 76.84, 95% CI = (10.78, 547.85), *p* < 0.0001, *I*^2^ = 0%], while the low dose had a significant but slightly lower improvement [RR = 55.44, 95% CI = (7.79, 394.62), *p* < 0.0001, *I*^2^ = 0%]. (Moderate certainty of evidence) (Figure 5).

#### 75% reduction in SALT score

The pooled estimate of two trials involving 1,209 patients included studies showing a substantial number of patients randomized to deuruxolitinib attaining a 75% reduction in SALT score [RR = 93.66, 95% CI = (23.42, 374.65), *p* < 0.00001, *I*^2^ = 0%] from baseline up to week 24. The high dose had more than one-hundred-fold number of patients achieving this criterion [RR = 105.74, 95% CI = (14.88, 751.50), *p* < 0.00001, *I*^2^ = 0%], while the low dose showed a significant number of improved patients but with trivial inferiority to the higher dose [RR = 82.97, 95% CI = (11.69, 589.05), *p* < 0.00001, *I*^2^ = 0%]. (High certainty of evidence) (Figure 6).

#### SPRO changes from baseline

One thousand one hundred and twelve patients randomized to deuruxolitinib in two RCTs reported a significant improvement in SPRO from baseline up to week 24 compared to the control [MD = −1.52, 95% CI = (−1.76, −1.27), *p* < 0.00001, *I*^2^ = 69%]. Both high and low doses had a significant number of participants that showed improvement in SPRO from baseline, respectively, [MD = −1.70, 95% CI = (−1.91, −1.49), *p* < 0.00001, *I*^2^ = 0%] [MD = −1.35, 95% CI = (−1.64, −1.06), *p* < 0.00001, *I*^2^ = 63%]. (High certainty of evidence) (Figure 7).

#### >2 points in SPRO

Patients randomized to deuroxolitinib (*n* = 1,209) in two RCTs were five times more likely to have an improvement of more than two SPRO points [RR = 4.98, 95% CI = (3.79, 6.54), *p* < 0.00001, *I*^2^ = 0%]. The high dose demonstrated the most superior effect [RR = 5.48, 95% CI = (3.51, 8.57), *p* < 0.00001, *I*^2^ = 35%], while the low dose had an attenuated significant effect [RR = 4.63, 95% CI = (2.95, 7.27), *p* < 0.00001, *I*^2^ = 36%]. (High certainty of evidence) (Figure 8).

### Safety outcome

#### Treatment-emergent adverse events of interest

Meta-analysis of the three included RCTs evaluated the safety of deuruxolitinib in patients with AA compared to placebo at week 28. The most common treatment-emergent adverse events (TEAEs) were grade 3 or 4 increase in blood creatinine phosphokinase (CPK) [RR = 2.79, 95% CI = (1.5, 4.99), *p* = 0.0006, *I*^2^ = 0%], acne (significant in the 12 mg dose only) [RR = 1.80, 95% CI = (0.84, 3.88), *p* = 0.13, *I*^2^ = 64%], and headache [RR = 1.49, 95% CI = (0.98, 6.54), *p* = 0.06, *I*^2^ = 44%]. The high-dose regimen recorded more significant increases in CPK [RR = 3.35, 95% CI = (1.46, 7.69), *p* = 0.004, *I*^2^ = 0%] than the low-dose regimen [RR = 2.33, 95% CI = (1.03, 5.29), *p* = 0.04, *I*^2^ = 0%] (High certainty of evidence), all of which were asymptomatic. Similarly, the incidence of acne was more significant with the higher dose [RR = 3.00, 95% CI = (1.62, 5.57), *p* = 0.0005, *I*^2^ = 0%] (High certainty of evidence) in comparison to the lower dose [RR = 1.00 95% CI = (0.26, 3.84), *p* = 0.99, *I*^2^ = 75%] (Moderate certainty of evidence). Regarding headache, the high dose regimen did not cause a significant incidence [RR = 1.33, 95% CI = (0.61, 2.89), *p* = 0.48, *I*^2^ = 62%], while the low dose caused more headaches but was at the borderline point of significance [RR = 1.66, 95% CI = (1, 2.78), *p* = 0.05, *I*^2^ = 32%]. (High certainty of evidence) (Figure 9).

#### Infections and infestations

The pooled estimate of 958 patients showed no significant increase in the incidence of upper respiratory tract infections (URTI) [RR = 0.59, 95% CI = (0.34, 1.03), *p* = 0.07, *I*^2^ = 15%] or nasopharyngitis [RR = 1.23, 95% CI = (0.82, 1.85), *p* = 0.31, *I*^2^ = 14%]. A dose-based subgroup analysis demonstrated that the placebo group has a significant increase in URTI cases versus the low dose [RR = 0.38, 95% CI = (0.18, 0.83), *p* = 0.01, *I*^2^ = 15%] (Low certainty of evidence). On the other hand, the high dose had no differences versus placebo [RR = 0.83, 95% CI = (0.40, 1.74), *p* = 0.63, *I*^2^ = 18%]. Neither high [RR = 1.53, 95% CI = [0.56, 4.16], *p* = 0.41, *I*^2^ = 57%] (High certainty of evidence) nor low [RR = 1.19, 95% CI = (0.74, 1.91), *p* = 0.47, *I*^2^ = 0%] (Moderate certainty of evidence) doses caused a significant increase in the incidence of nasopharyngitis (Figure 10).

## Discussion

This systematic review and meta-analysis evaluated the efficacy and safety of deuruxolitinib for moderate-to-severe AA as monotherapy. Three RCTs were enrolled for analysis with a total of 1,372 participants. Deuruxolitinib, a JAK1/JAK2 inhibitor, showed a significant improvement and hair regrowth in terms of all efficacy outcomes, which supports the theory that it can be a promising treatment option for AA. The safety profile demonstrated that adverse events including increased levels of CPK and acne were the most commonly reported TEAEs, particularly with a higher dose, while deuruxolitinib has no relation to more infections or infestations.

Topical or systemic steroid therapy is considered the first line and most widely used treatment for AA. It is a highly effective drug and can be combined with other topical treatments including minoxidil and glycopyrrolate. In more severe cases of AA, systemic treatments may be administered including diphenylcyclopropenone and immunosuppressants (21). However, treating severe AA can be a significant therapeutic challenge. The currently available therapeutic options, including steroids, minoxidil, anthralin, tacrolimus, or cryotherapy, have no strong evidence supporting their ongoing effectiveness in extensive AA either due to their minimal efficacy and/or side effects (28–30). As AA is an autoimmune disease associated with complex overexpression of different cytokines, the JAK/STAT signalling pathway plays a huge role and acts as the intersection for different inflammatory factors. Thus, JAK inhibitors provide the benefits of both significant efficacy and minimal side effects (22, 23). Multiple JAK inhibitors have been used and tested for the treatment of AA. In 2022, baricitinib, a JAK1/2, became the first JAK inhibitor that received FDA approval for the treatment of AA, followed by ritlecitinib, a JAK3, a year later (24, 25). The efficacy and safety of JAK inhibitors have been the subject of several published systematic reviews. A previously published report demonstrated the superiority of oral JAK inhibitors over the control group in terms of hair regrowth and response rate (15). The results of other studies showed that JAK inhibitors including baricitinib, ritlecitinib, brepocitinib, ruxolitinib, and tofacitinib were associated with more hair regrowth and lower SALT scores in comparison to placebo (26, 27). Moreover, a recent systematic review and meta-analysis that included a study assessing deuruxolitinib concluded that baricitinib and deuruxolitinib appear to be superior to other JAK inhibitors (6). Considering its efficacy, deuruxolitinib is expected to follow baricitinib and ritlecitinib about FDA approval. Notably, the reported risk ratios in our study for some efficacy outcomes, such as achieving a 75 and 90% reduction in SALT score were very high (RR = 93.66 and RR = 65.26, respectively). These elevated values are likely due to the very low response rate in the placebo groups, as opposed to the substantial clinical efficacy observed in the treatment arms (i.e. achieving the clinical improvement cut-off).

Included trials examined the effect of 4, 8, and 12 mg BID deuruxolitinib regimens versus placebo. However, the most recent trials only examined 8 and 12 mg BID because 4 mg demonstrated a suboptimum effect, with only 21.4% of patients achieving at least a 50% reduction in SALT score compared to the higher-dosed regimens 47.4 and 58.3% respectively, and placebo (9.3%). Safety-wise, the lower dose (4 mg BID), paradoxically, had the highest percentage of patients achieving at least one adverse event (86.4%) while 8 mg recorded at 81.6%, 12 mg BID at 83.3%, and the placebo arm at 70.5%. The lower dose had the highest incidence of specific AEs including nausea, vomiting, and cough. These findings are likely the reason this regimen (4 mg BID) was discontinued in later clinical studies, and, consequently, was not included within the subgroup of our meta-analysis due to the lack of a pooled estimate. This is most in keeping with the finding that headache is more significant within patients randomized to 8 mg BID (RR = 1.66) rather than 12 mg BID (RR = 1.33) despite its statistical insignificance (*p* = 0.05) at the cut-off point and the statistical heterogeneity (*I*^2^ = 76%) within the 12 mg subgroup. This raises more questions concerning an inverse dose–response relationship to certain adverse events, similar to the aforementioned point regarding the paradoxical effect of the lower dose. This, however, cannot be stated with regards to placebo causing more URTI than 8 mg BID of deuruxolitinib. Some JAK inhibitors have a well-documented effect of causing increased URTIs, making this estimate more likely to result out of chance. Thus, it was regarded to have a low certainty of evidence upon GRADE assessment.

This paper is the first dedicated systematic review to use a meta-analysis to compare deuruxolitinib versus placebo for patients with moderate-to-severe AA. Though the novelty of this topic and the high quality of included RCTs are key strengths, so too are other traits including the lack of conflict of interest among authors, the in-depth qualitative analysis of the safety profile, and the dose-based subgroup analysis. These remain vital strengths of our study, especially within the context of TEAEs of interest. Another supporting aspect is that pooled effects of efficacy had a nearly unified inclusion and exclusion criteria between included RCTs and an identical timepoint of efficacy observation at week 24, eliminating any confounding effects arising from these factors. Lastly, the evaluation of certainty of evidence of all pooled estimates, including subgroups, adds a considerable amount to the literature about the effects of different regimens and dose-related efficacy and safety outcomes.

According to the Cochrane Handbook for Systematic Reviews of Interventions, *I*^2^ ranging from 30 to 60% is considered moderate heterogeneity. Within our quantitative synthesis, few estimates were found to be heterogeneous. Within these cases, it is not feasible to investigate the roots of this heterogeneity due to having a minimal number of studies (two studies) within a forest plot or a subgroup from which to generate a pooled estimate: This is a limitation on its own. Also, there is a lack of a hypothesis from which to investigate the outcomes with more than two studies using a sensitivity analysis and an absence of clinical context, as most of these outcomes extremely favour deuruxolitinib over placebo. Therefore, this heterogeneity is only a statistical heterogeneity. Another limitation is that funnel plots were not generated. This is due to the latest recommendations regarding having less than ten studies included in a quantitative analysis. The most important limitation is the possible conflict of interest, as all included studies were done in the early developmental stages of the drug, and therefore conducted by the developing and manufacturing company or its affiliates. Industry funding can sometimes affect the way outcomes are reported or emphasized; however, the trials included in our review maintained rigorous methodologies, clear outcome definitions, and consistent reporting standards.

## Data Availability

The datasets presented in this study can be found in online repositories. The names of the repository/repositories and accession number(s) can be found in the article/Supplementary material.
