# Supplementary material for: Evaluation of the efficacy and treatment-emergent adverse events of deuruxolitinib for moderate to severe alopecia areata: a dose-ranging meta-analysis of 1,372 randomized patients
Source: Front Med (Lausanne). 2025 Oct 7;12:1641245. doi: 10.3389/fmed.2025.1641245 (PMC12539349; doi:10.3389/fmed.2025.1641245)
Supplement: Supplementary file 1 [file Table_1.docx]

|  | **Certainty assessment** | | | | | | | | **Certainty** |
| --- | --- | --- | --- | --- | --- | --- | --- | --- | --- |
| **Outcome** | | **Regimen** | **Study design** | **Risk of bias** | **Inconsistency** | **Indirectness** | **Imprecision** | **Other considerations** |  |
| **Relative change in SALT score from baseline** | | 8 mg BID | randomised trials | not serious | not serious | not serious | not serious | Very strong association | ⨁⨁⨁⨁ High |
|  |  | 12 mg BID | randomised trials | not serious | not serious | not serious | not serious | Very strong association | ⨁⨁⨁⨁ High |
| **Number of patients achieving SALT90** | | 8 mg BID | randomised trials | not serious | not serious | not serious | Serious^a^ | Very strong association | ⨁⨁⨁⨁ High |
|  |  | 12 mg BID | randomised trials | not serious | not serious | not serious | Serious^a^ | Very strong association | ⨁⨁⨁⨁ High |
| **Number of patients achieving SALT75** | | 8 mg BID | randomised trials | not serious | not serious | not serious | Serious^a^ | Very strong association | ⨁⨁⨁⨁ High |
|  |  | 12 mg BID | randomised trials | not serious | not serious | not serious | Serious^a^ | Very strong association | ⨁⨁⨁⨁ High |
| **Number of patients achieving ≥ 2 points improvement in SPRO score** | | 8 mg BID | randomised trials | not serious | not serious | not serious | not serious | Strong association | ⨁⨁⨁⨁ High |
|  |  | 12 mg BID | randomised trials | not serious | not serious | not serious | not serious | Strong association | ⨁⨁⨁⨁ High |
| **Change in SALT score from baseline** | | 8 mg BID | randomised trials | not serious | not serious | not serious | not serious | Very strong association | ⨁⨁⨁⨁ High |
|  |  | 12 mg BID | randomised trials | not serious | not serious | not serious | not serious | Very strong association | ⨁⨁⨁⨁ High |

#### Explanations

1. Wide confidence interval
2. High heterogeneity

|  | **Certainty assessment** | | | | | | | | **Certainty** |
| --- | --- | --- | --- | --- | --- | --- | --- | --- | --- |
| **Outcome** | | **Regimen** | **Study design** | **Risk of bias** | **Inconsistency** | **Indirectness** | **Imprecision** | **Other considerations** |  |
| **Increased blood creatinine phosphokinase** | | 8 mg BID | randomised trials | not serious | not serious | not serious | not serious | none | ⨁⨁⨁⨁ High |
|  |  | 12 mg BID | randomised trials | not serious | not serious | not serious | Serious^a^ | strong association | ⨁⨁⨁⨁ High |
| **Acne** | | 8 mg BID | randomised trials | not serious | Serious^b^ | not serious | not serious | none | ⨁⨁⨁◯  Moderate |
|  |  | 12 mg BID | randomised trials | not serious | not serious | not serious | not serious | strong association | ⨁⨁⨁⨁ High |
| **Headache** | | 8 mg BID | randomised trials | not serious | not serious | not serious | not serious | none | ⨁⨁⨁⨁ High |
|  |  | 12 mg BID | randomised trials | not serious | not serious | not serious | not serious | none | ⨁⨁⨁⨁ High |
| **Upper respiratory tract infections** | | 8 mg BID | randomised trials | not serious | not serious | Very serious^c^ | not serious | none | ⨁⨁◯◯ Low |
|  |  | 12 mg BID | randomised trials | not serious | not serious | not serious | not serious | none | ⨁⨁⨁⨁ High |
| **Nasopharyngitis** | | 8 mg BID | randomised trials | not serious | not serious | not serious | not serious | none | ⨁⨁⨁⨁ High |
|  |  | 12 mg BID | randomised trials | not serious | Serious^b^ | not serious | not serious | none | ⨁⨁⨁◯  Moderate |

1. Wide confidence interval
2. High heterogeneity
3. Inexplicable relation between placebo and the incidence of upper respiratory tract infections
