# Supplementary material for: Evaluation of the efficacy and treatment-emergent adverse events of deuruxolitinib for moderate to severe alopecia areata: a dose-ranging meta-analysis of 1,372 randomized patients
Source: Front Med (Lausanne). 2025 Oct 7;12:1641245. doi: 10.3389/fmed.2025.1641245 (PMC12539349; doi:10.3389/fmed.2025.1641245)
Supplement: Supplementary file 2 [file Supplementary_file_1.docx]

**The Cochrane Central Register of Controlled Trials (CENTRAL) Search Strategy**

ID Search Hits

#1 MeSH descriptor: [Alopecia Areata] explode all trees 432

#2 ("alopecia areata"):ti,ab,kw OR (AA):ti,ab,kw OR (alopecia):ti,ab,kw (Word variations have been searched) 11843

#3 #1 OR #2 11843

#4 (ctp-543):ti,ab,kw OR (deuruxolitinib):ti,ab,kw (Word variations have been searched) 14

#5 #3 AND #4 9

Search results = 9

**Medline (PubMed) Search Strategy:**

| **Search number** | **Query** | **Sort By** | **Filters** | **Search Details** | **Results** |
| --- | --- | --- | --- | --- | --- |
| **10** | (((("alopecia"[MeSH Terms]) OR ("alopecia areata"[MeSH Terms])) OR ("alopecia"[All Fields])) OR ("alopecia areata"[All Fields])) AND (("deuruxolitinib"[All Fields]) OR ("ctp 543"[All Fields])) |  | Randomized Controlled Trial | (("alopecia"[MeSH Terms] OR "alopecia areata"[MeSH Terms] OR "alopecia"[All Fields] OR "alopecia areata"[All Fields]) AND ("deuruxolitinib"[All Fields] OR "ctp 543"[All Fields])) AND (randomizedcontrolledtrial[Filter]) | 2 |
| **9** | (((("alopecia"[MeSH Terms]) OR ("alopecia areata"[MeSH Terms])) OR ("alopecia"[All Fields])) OR ("alopecia areata"[All Fields])) AND (("deuruxolitinib"[All Fields]) OR ("ctp 543"[All Fields])) |  |  | ("alopecia"[MeSH Terms] OR "alopecia areata"[MeSH Terms] OR "alopecia"[All Fields] OR "alopecia areata"[All Fields]) AND ("deuruxolitinib"[All Fields] OR "ctp 543"[All Fields]) | 14 |
| **8** | ("deuruxolitinib"[All Fields]) OR ("ctp 543"[All Fields]) |  |  | "deuruxolitinib"[All Fields] OR "ctp 543"[All Fields] | 16 |
| **7** | "ctp 543"[All Fields] |  |  | "ctp 543"[All Fields] | 6 |
| **6** | "deuruxolitinib"[All Fields] |  |  | "deuruxolitinib"[All Fields] | 13 |
| **5** | ((("alopecia"[MeSH Terms]) OR ("alopecia areata"[MeSH Terms])) OR ("alopecia"[All Fields])) OR ("alopecia areata"[All Fields]) |  |  | "alopecia"[MeSH Terms] OR "alopecia areata"[MeSH Terms] OR "alopecia"[All Fields] OR "alopecia areata"[All Fields] | 28,765 |
| **4** | "alopecia areata"[All Fields] |  |  | "alopecia areata"[All Fields] | 6,424 |
| **3** | "alopecia"[All Fields] |  |  | "alopecia"[All Fields] | 28,563 |
| **2** | "alopecia areata"[MeSH Terms] |  |  | "alopecia areata"[MeSH Terms] | 4,451 |
| **1** | "alopecia"[MeSH Terms] |  |  | "alopecia"[MeSH Terms] | 17,515 |

Search results = 2

Clinicaltrials.gov

**Condition/Disease**

Alopecia Areata OR Alopecia

**Intervention/Treatment**

CTP-543 OR Deuruxolitinib

**Limiters**

Study results: with results

Search results = 6
